# Cancer Mortality among Asians and Pacific Islanders in New York City, 2001–2010

**DOI:** 10.1155/2013/986408

**Published:** 2013-12-12

**Authors:** Vivian Huang, Wenhui Li, Josephine Tsai, Elizabeth Begier

**Affiliations:** New York City Department of Health and Mental Hygiene, 125 Worth Street, 2nd Floor, Room WS 2-14, New York, NY 10013, USA

## Abstract

Asians and Pacific Islanders' (APIs) leading cause of death is cancer. We compared APIs' age-adjusted cancer mortality rates to other racial/ethnic groups and by API subgroup (i.e., Chinese, Koreans, Asian Indians, and Filipinos) using New York City (NYC) Mortality data and Census Bureau population estimates for 2001–2010. While other racial/ethnic groups' overall cancer mortality rates declined in NYC during the last decade, APIs remained stable. APIs overall had the lowest mortality rates for more common cancer types (i.e., lung, colorectal, breast, and prostate), but the highest mortality rates for certain less common cancers (i.e., nasopharyngeal, stomach, and liver). Chinese New Yorkers' lung cancer death rates were very high compared to other APIs and comparable to non-Hispanic whites (47.1/100,000 versus 49.5/100,000, resp.). Chinese men had much higher nasopharyngeal cancer mortality rates (4.5/100,000 versus 0.3/100,000 for non-Hispanic whites). Korean men had the highest liver and stomach cancer mortality rates (25.3/100,000 and 27.7/100,000, resp., versus 7.9/100,000 and 6.0/100,000 for non-Hispanic whites). Analysis of cancer rates by API subgroup provides the detailed information needed to plan cancer prevention efforts. These findings warrant consideration of targeted cancer mortality prevention efforts for affected subgroups, including hepatitis vaccination, screening, and treatment; smoking cessation; and cancer screening.

## 1. Introduction

Asians and Pacific Islanders (APIs) constitute 5% of the United States (US) population and 13% of the New York City (NYC) population [1]. From 2000 through 2010, the API population experienced the fastest rate of growth than any other racial/ethnic groups both nationally (43% increase) and in NYC (32% increase) [2]. The city's API population is the largest among US cities, 1.1 million persons, according to the 2010 Census.

While cardiovascular disease is the leading cause of mortality among non-Hispanic whites, non-Hispanic blacks, and Hispanics, cancer is the leading cause of death among APIs, both nationally in 2007 (106.7 per 100,000) and in NYC in 2010 (105.9 per 100,000) [3, 4]. APIs experience lower death rates for certain common cancers (i.e., lung, colorectal, breast, and prostate) than other racial/ethnic groups in the United States [5]. However, APIs experience the highest death rates from some less common cancers, particularly those associated with infectious agents, such as nasopharynx, liver, and stomach cancer [6].

Published analyses on cancer mortality among APIs have largely focused on California [7, 8] and New York City [9, 10], except one study utilizing data from selected sites throughout the US [11], but all these studies are based on data that is more than a decade old. In California, McCracken et al. examined cancer mortality among Asian Americans overall from 2000 to 2002 [7], and Kwong et al. described cancer mortality by Asian subgroups from 1997 through 2001 [8]. In NYC, Freeman et al. examined cancer mortality by overall racial/ethnic group between 1990 and 2000 [9]. Miller et al. examined cancer incidence and mortality by Asian subgroups for 1998–2002 from selected state and regional cancer registries covering 54% of the US API population [11]. Cancer mortality among API subgroups has not been studied in NYC, aside from a 20-year-old NYC study (1988 through 1992) examining site-specific cancer mortality rates among the Chinese only [10]. Thus, to our knowledge, no recent analyses of API or API subgroup cancer mortality rates within a US population have been conducted. We aim to characterize site-specific cancer mortality rates among APIs in NYC, comparing them to other racial/ethnic groups for the 10-year period from 2001 through 2010. We will also examine site-specific cancer rates among different API subgroups (i.e., Chinese, Korean, Asian Indian, and Filipino) to explore heterogeneity within the diverse API group.

## 2. Materials and Methods

### 2.1. Data Sources

This analysis was based on New York City annual population estimates obtained from the US Census Bureau, the American Community Survey Public Use Microdata Samples (2005 through 2009), and mortality data for the 10-year period (2001 through 2010) from the New York City Department of Health and Mental Hygiene's (NYC DOHMH) Bureau of Vital Statistics.

### 2.2. Mortality Data

Deidentified mortality data were obtained. Variables included sex, age, race/ethnicity, ancestry, birthplace, education, marital status, and underlying cause of death. Underlying causes of death were coded according to the 10th Revision of the *International Statistical Classification of Diseases and Related Health Problems* (ICD-10) [12]. Common site-specific cancers across all racial/ethnic groups (i.e., lung, colon and rectum, esophagus, prostate, breast, ovary, and cervix) and those specifically common in APIs (i.e., nasopharynx, liver, and stomach) were selected for study. API subgroups were determined by the races reported on death certificates, which is provided by funeral directors in NYC. We did not differentiate between foreign-born versus US-born APIs because the proportion of US-born was only 2.1% of deaths which is too small to merit analysis. This is likely because of the relatively young age of New York City's US-born Asian population; 81% are less than 45 years of age based on US Census Bureau's 2005–2009 American Community Survey Microdata Sample.

### 2.3. Calculation of Age-Adjusted Cancer Mortality Rates

Cancer mortality rates and 95% confidence intervals were calculated for major racial/ethnic groups using NYC DOHMH mortality data and US Census population estimates. Cancer mortality rates and 95% confidence intervals for Chinese, Korean, Asian Indian, and Filipino subgroups were calculated using NYC DOHMH mortality records and the 2005–2009 American Community Survey Public Use Microdata Sample. The 2010 US Census reports information on 6 Asian subgroups: Chinese, Korean, Asian Indian, Filipino, Japanese, and Vietnamese. New York City's Japanese and Vietnamese populations were too small to allow for inclusion in this analysis. All rates were age adjusted and annualized [13].

### 2.4. Data Analysis

We used SAS statistical package version 9.2 for data management and statistical analysis.

## 3. Results

**Figure 1:**
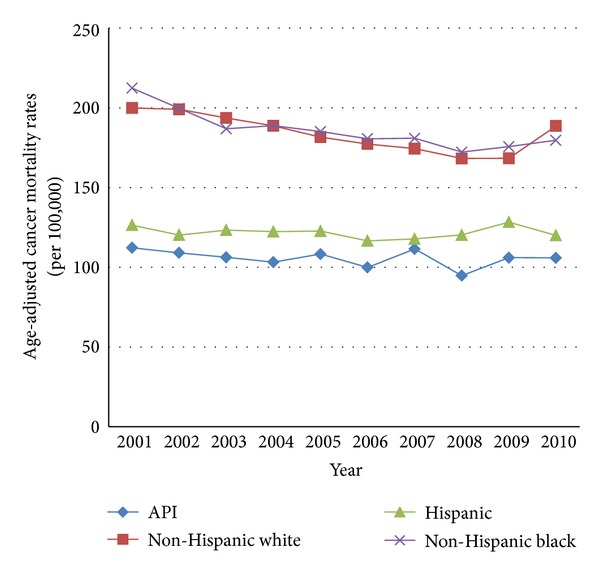
Mortality trends, New York City, 2001–2010 malignant neoplasms.

In 2010, 13,333 (25.4%) New York City decedents died from a malignant neoplasm. Among API decedents, 943 (29.9%) died from a malignant neoplasm. APIs have the overall lowest cancer mortality rates across all racial/ethnic groups. However, between 2001 and 2010, cancer mortality rates declined in all racial/ethnic groups in NYC, except APIs. The API cancer mortality rate has remained stable from 2001 through 2010 (see Figure 1).

### 3.1. Decedent Characteristics by Racial/Ethnic Groups

From 2001 through 2010, a total of 82,608 decedents died in New York City from the 10 selected cancers (Table 4); 5,548 (6.7%) of these decedents were APIs (3,200 males and 2,348 females) (Table 1). Compared with non-Hispanic whites, API decedents were more likely to be male, married, and having less than a high school education. On average, API decedents died 5 years earlier from cancer than did non-Hispanic whites.

### 3.2. Site-Specific Cancer Mortality by Racial/Ethnic Groups and Sex

The age-adjusted nasopharyngeal cancer mortality rate among API men (2.6 per 100,000) was the highest across all racial/ethnic groups and nearly 10 times higher than the rate for non-Hispanic white men (0.29 per 100,000; see Table 2). The mortality rates of liver and stomach cancer were twice as high in API men as in non-Hispanic white men (liver cancer, 15.9 versus 7.9 per 100,000 and stomach cancer, 11.8 versus 6.0 per 1000,000, resp.).

The mortality rate for nasopharyngeal cancer among API women (0.7 per 100,000) was the highest of all racial/ethnic groups, 7 times as high as in non-Hispanic white women (0.1 per 100,000). The liver cancer mortality rate was almost 2 times higher in API women (5.4 per 100,000) than in non-Hispanic white women (3.0 per 100,000), and API women had the highest stomach cancer mortality rate (5.6 per 100,000) of all racial/ethnic groups.

Among both men and women, APIs have the lowest colorectal cancer mortality rates of all racial/ethnic groups. API men and women have age-adjusted lung cancer mortality rates (38.2 and 16.7 per 100,000, resp.) that surpass those of Hispanic men and women (33.5 and 14.7 per 100,000, resp.) but not non-Hispanic whites or blacks. For all studied sites, cancer death rates were higher in API men than API women.

### 3.3. Trends in Cancer Mortality across Racial/Ethnic Groups

**Figure 2:**
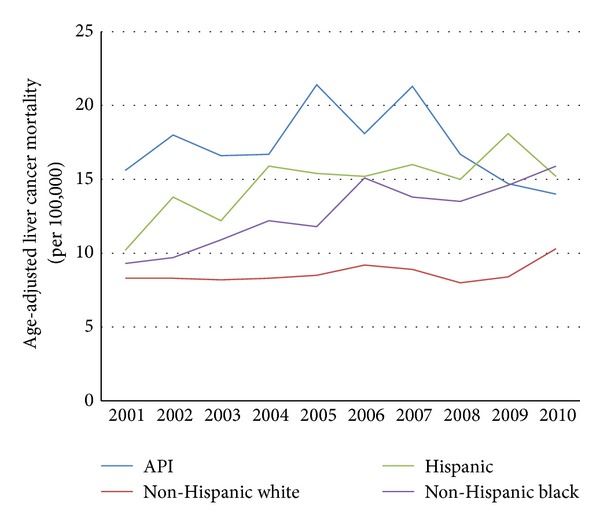
Trends in male age-adjusted liver cancer mortality rates by ethnicity, 2001–2010.

**Figure 3:**
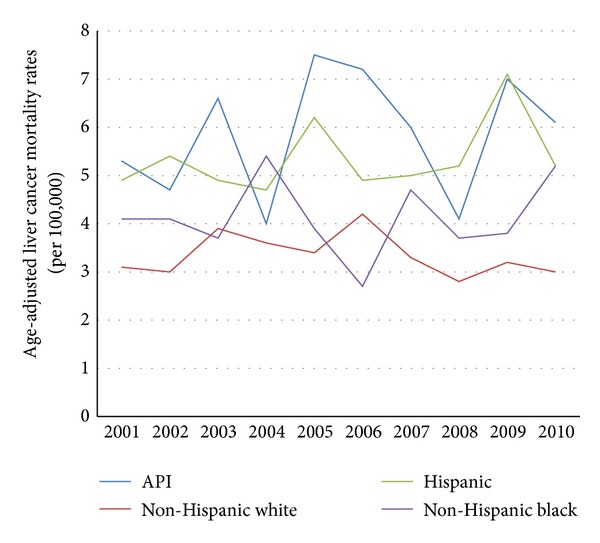
Trends in female age-adjusted liver cancer mortality rates by ethnicity, 2001–2010.

**Figure 4:**
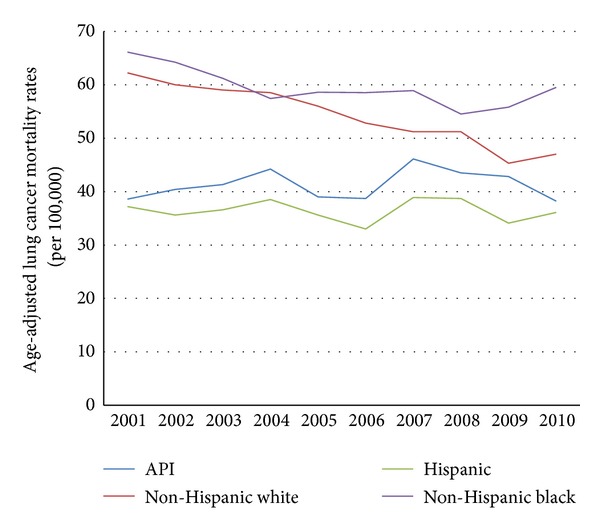
Trends in male age-adjusted lung cancer mortality rates by ethnicity, 2001–2010.

**Figure 5:**
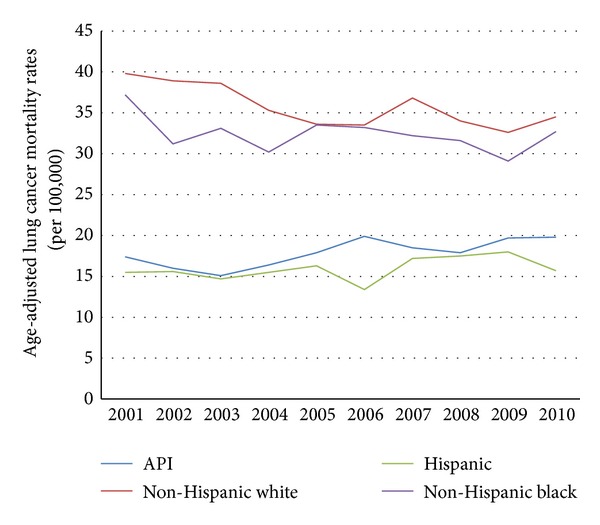
Trends in female age-adjusted lung cancer mortality rates by ethnicity, 2001–2010.

From 2001 through 2010, API males experienced higher age-adjusted liver cancer mortality rates than non-Hispanic white males (15.6 to 14.0 per 100,000 versus 8.3 to 10.3 per 100,000, resp.; see Figure 2). From 2001 through 2010, API females experienced a slight increase in age-adjusted liver cancer mortality rates, while this rate was stable among non-Hispanic white females (5.3 to 6.1 per 100,000 versus 3.1 to 3.0 per 100,000, resp.; see Figure 3). In the last decade, API males did not experience the dramatic decline in age-adjusted lung cancer mortality rates that non-Hispanic white males did (38.6 to 38.2 per 100,000 versus 62.2 to 47.0 per 100,000, resp.; see Figure 4). Age-adjusted lung cancer mortality rates among API females increased during 2001 through 2010, while non-Hispanic white females experienced a decrease (17.4 to 19.7 per 100,000 versus 39.8 to 34.5 per 100,000, resp.; see Figure 5).

### 3.4. Decedent Characteristics by API Subgroups

Overall, 5,548 cancer deaths occurred among APIs in New York City from 2001 through 2010 (see Table 3). Chinese comprise the majority (66.7%) of API cancer decedents. Compared to other API subgroups, Chinese decedents were more likely to be male, have less than a high school education, and die at an older age on average. Compared to other API subgroups, Asian Indian cancer decedents were more likely to be married and die at a younger age. Filipino decedents were more likely to be female and have more than a high school education.

### 3.5. Cancer Mortality by API Subgroups

#### 3.5.1. Chinese

Chinese men and women had the highest lung cancer mortality rates (47.1 and 20.0 per 100,000, resp.) as compared to other API subgroups. Lung cancer mortality rates in the Chinese have increased from 2001 to 2010 (40.0 to 51.5 per 100,000 in men and 16.2 to 25.8 per 100,000 in women), while they have decreased in non-Hispanic whites (62.2 to 47.0 per 100,000 in men and 39.8 to 34.5 per 100,000 in women). Among APIs, Chinese women had the highest colon cancer mortality rate (10.6 per 100,000) among women and Chinese men had the second highest colon cancer mortality rate (16.1 per 100,000) among men. Chinese men and women had the highest nasopharyngeal cancer mortality rates (4.5 and 1.2 per 100,000, resp.) across all racial/ethnic groups, and these rates were statistically significantly higher than in the other racial/ethnic groups.

#### 3.5.2. Korean

Korean men had the highest liver and colon cancer rates (25.3 and 17.6 per 100,000, resp.) compared to other API subgroups. Korean women had the highest liver cancer mortality rate, which was statistically significantly higher than other API subgroups (7.5 per 100,000 versus 6.0 per 100,000 for Chinese women, 2.9 per 100,000 for Asian Indian women and 2.6 per 100,000 for Filipino women). Korean men and women had the highest stomach cancer rates, which were statistically significantly higher than other API subgroups (27.7 and 11.0 per 100,000, resp.). Korean men and women had the second highest age-adjusted lung cancer mortality rates (38.9 and 12.9 per 100,000, resp.).

#### 3.5.3. Filipino

Among API subgroups, Filipino men had the highest prostate mortality rate (9.5 per 100,000). Filipino women had the highest breast and ovarian cancer mortality rates (13.4 and 6.4 per 100,000, resp.).

## 4. Discussion

We investigated site-specific cancer mortality rates among APIs and API subgroups, given that cancer is the leading cause of death among APIs. While APIs in NYC have the lowest overall cancer mortality rate of the 4 major racial/ethnic groups, certain site-specific cancer rates are much higher among APIs than other racial/ethnic groups and among API subgroups. Examining rates for APIs as a group masks the true heterogeneity of their cancer burden. Varying environmental and behavioral influences, including tobacco use, exposure to infectious agents, and diet, are likely driving these observed patterns of API cancer mortality. These findings warrant consideration of targeted cancer mortality prevention efforts for affected subgroups, including hepatitis vaccination, screening, and treatment; smoking cessation; and cancer screening.

### 4.1. Lung Cancer

Lung cancer death rates in Chinese New Yorkers are high and have increased from 2001 through 2010 for both men and women. It is known that tobacco smoking, primarily cigarettes, is the major cause of lung cancer. In NYC, the 2010 Community Health Survey, a NYC DOHMH population-based telephone survey, found a smoking prevalence of 20% among Chinese men and less than 1% among Chinese women. The increasing lung cancer death rate among Chinese women in the setting of low prevalence of smoking is worrisome, and more research is needed to determine factors contributing to the increased lung cancer death rates in Chinese women. Second-hand smoke exposure at home and work and exposure to cooking oil from high-temperature frying may contribute to the increased risk of lung cancer in Chinese women [14]. In addition, the smoking prevalence among the Chinese population overall has remained the same over 8 years (9.2% in 2002 and 10.2% in 2010), whereas the overall smoking prevalence in NYC dropped significantly from 21.5% in 2002 to 14% in 2010. Given that NYC mounted an aggressive tobacco control program during this period, the increasing lung cancer death rates and the stable smoking prevalence rates among the Chinese suggest that we may not be reaching this subgroup.

Global data suggest that lung cancer mortality rates in China are lower than in NYC. It is not known why this is true, but two possibilities would be increased immigration to NYC from areas of China with higher rates of lung cancer and decreased accuracy of cause of death information. In 2008, according to the International Agency for Research on Cancer, lung cancer mortality rates were 39.6 per 100,000 in Chinese men and 18.3 per 100,000 in Chinese women [15] versus 47.1 and 20.0 per 100,000 for Chinese men and women in NYC, respectively, per our analyses.

One reason that could explain these persistently high rates of lung cancer mortality among the Chinese in NYC is the culturally accepted nature of tobacco smoking in certain Asian countries, particularly among men. According to the Global Adult Tobacco Survey, 63% of men in China currently smoked in 2010 [16]. In addition, giving cigarettes as gifts during special occasions is a cultural norm [17], and immigrants have access to low-cost cigarettes from Taiwan and China.

Since 2005, the NYC Mayor's Office and NYC DOHMH have launched several culturally relevant media campaigns focusing on the Chinese, including translated posters and print advertisements to promote tobacco cessation. The NYC DOHMH has a long-standing nicotine patch and gum give-away program [18], and in 2010, online registration was made available in Chinese along with translated nicotine patch and gum kit contents. DOHMH also has a 311 hotline with Chinese language capability to help interested callers quit smoking. In 2012, a dedicated Chinese hotline was created to attract more participants to the nicotine patch and gum give-away program. Despite these efforts, the prevalence of smoking in Chinese New Yorkers has not changed substantially. Hopefully, these recent interventions will show an impact in future assessments. More research is needed to determine the barriers to quitting smoking in the Asian population.

Closely following the Chinese, Koreans have the second highest lung cancer mortality rates (38.9 per 100,000 for men and 12.9 per 100,000 for women) among APIs. In South Korea from 2008 to 2010, 42.3% of Korean men have been reported to smoke [19]. In NYC, for 2006–2008, Korean's self-reported rate of smoking was 16.9% based on the NYC Community Health Survey, although small numbers limit the precision of that estimate. NYC has not focused on special tobacco cessation efforts in this group due to its small population size (only 1.0% of NYC population).

### 4.2. Nasopharyngeal Cancer

Nasopharyngeal cancer death rates in NYC are higher among APIs than other racial/ethnic groups, and this cancer disproportionately affects the Chinese. The nasopharyngeal cancer mortality rate in Chinese men (4.5 per 100,000) was 15 times that of non-Hispanic whites (0.3 per 100,000). In Chinese women, the nasopharyngeal cancer mortality rate (1.2 per 100,000) was 12 times as high as in non-Hispanic white women (0.1 per 100,000). According to the World Health Organization, nasopharyngeal cancer mortality rates in 2008 in China for males and females were 1.9 and 0.8 per 100,000, respectively, based on data from the International Agency for Research on Cancer [20]. Death rates in the US may be higher due to immigration patterns. Most immigrants from China to the US come from Guangdong Province, where the incidence of nasopharyngeal cancer is very high (25 cases per 100,000) [21]. Several epidemiologic studies have proposed that the Epstein-Barr virus (EBV), smoked fish products, and genetic predisposition are associated with high nasopharyngeal cancer rates among the Chinese [22, 23]. In certain parts of southern China, particularly Guangdong Province where nasopharyngeal cancer is common, at-risk individuals are being screened by EBV testing, followed by periodic examination of the nasopharynx and neck if positive [24]. Earlier detection of nasopharyngeal carcinoma allows for treatment with improved 5-year survival [25]. In the United States, no nasopharyngeal cancer screening guidelines exist. In cities like NYC where a large proportion of Chinese immigrants are from southern China, a high index of clinical suspicion for this type of cancer should be maintained by clinicians caring for these immigrants. Further research is indicated to evaluate whether the screening approach used in southern China is effective at preventing mortality and to determine whether screening is appropriate for Chinese New Yorkers.

### 4.3. Liver Cancer

In NYC, the liver cancer death rate is higher among APIs, particularly Koreans and Chinese, than in other racial/ethnic groups. Hepatitis B virus (HBV) infection is highly prevalent in Asian countries, including South Korea and China, and is the main risk factor for developing liver cancer in countries endemic with HBV [26]. Other contributing risk factors include cirrhosis (associated with infection, obesity, and alcohol) and aflatoxin exposure [27]. NYC DOHMH surveillance for chronic HBV infection from June 2008 to November 2009 identified 156 cases of infection, of whom 87 (56%) were in Chinese born persons and few (<3) were in persons originating from Korea [28]. However, a 2008 prevalence study found that Koreans represented 4.6% of NYC's chronic HBV infections, while they constituted only 1.0% of the NYC population [29], confirming their higher risk for chronic infection compared to other New Yorkers. In one 2005 NYC study of a clinic-based screening program, Chinese had the highest prevalence (21%) of chronic HBV infection among the patients screened, but Koreans also had a high prevalence (5%) of infection [30]. Given elevated rates of liver cancer among the Chinese and Koreans, NYC clinicians should be aware that the Centers for Disease Control and Prevention and DOHMH currently recommend screening individuals at risk for HBV infection, including persons from HBV-endemic countries such as Korea and China, treatment of chronic HBV infection, and periodic liver cancer screening in those with chronic HBV infection, which may help to prevent progression and death from liver cancer [31].

Given that current evidence suggests that Koreans have a lower chronic HBV infection rate compared with Chinese, other factors may be playing a role in elevating their liver cancer death rate above that of the Chinese. When liver cancer was listed as the underlying cause of death, the use of alcohol as a contributing cause was mentioned in more Korean decedents (1.8%) than Chinese decedents (0.2%), although its mention was infrequent for both. In NYC's Community Health Survey for the period 2002 to 2008, individuals of Korean descent reported binge drinking almost 4 times more frequently than individuals of Chinese descent (23.5% versus 5.8%, resp.) and heavy drinking was reported more frequently as well (3.4% versus 1.3%), although small numbers limit the precision of these later estimates. Synergism between alcohol consumption and chronic HBV infection may possibly increase the risk of cirrhosis and thus liver cancer deaths in Koreans in NYC. Further research is needed to determine the contribution of alcohol use to liver cancer deaths among Koreans.

### 4.4. Stomach Cancer

APIs are at elevated risk for stomach cancer mortality, particularly Koreans and Chinese. *Helicobacter pylori* is thought to be an important risk factor for stomach cancer [32]. In addition, stomach cancer has also been associated with eating preserved food and foods rich in nitrites and/or nitrates [33]. No routine stomach cancer screening is recommended in the US, but screening programs have been implemented in Japan. The risk of stomach cancer deaths in the Japanese living in Japan (20.5 per 100,000 in men) is comparable to mortality rates in NYC Chinese (12.9 per 100,000 in men) and Koreans (27.7 per 100,000 in men). An improving trend in the stomach cancer survival rate has been observed in Japan and can possibly be attributed to early cancer detection [34]. A formal evaluation of the risk and benefits of using the Japanese screening approach in high-risk groups should be considered for NYC and other urban areas with a sizeable API population.

### 4.5. Breast Cancer

Breast cancer is the leading cause of cancer death in NYC Asian Indian and Filipina women. Multiple factors contribute to breast cancer mortality, including genetic predisposition and socioeconomic and cultural circumstances [35], but late-stage diagnosis is strongly associated with higher mortality rates. In the NYC Community Health Survey, women of Asian descent aged 40 years and older reported a lower rate of mammography screening (67.5%) than NYC women overall (75.3%), and specifically, Asian Indian women in NYC had a substantially lower rate of screening (58.9%). The rate for Filipina women was a few percent below the citywide rate (72.1%) based on their self-reported responses. However, a Hawaiian study found that Filipina women are less compliant with mammography guidelines and are diagnosed at a more advanced stage of cancer at time of diagnosis [36]. Efforts to target specific racial/ethnic groups to increase breast cancer screening may be needed. More research is also needed in NYC to determine the reasons for lower rates of mammography screening in Asian Indian and possibly Filipina women, which may contribute to higher breast cancer mortality rates.

### 4.6. Limitations

The limitations of this study include potential underascertainment of API population size by the 2010 Census or American Community Survey, which would result in an overestimation of mortality rates in the population. In particular, undocumented immigrants may not be captured in the census because of their reluctance to be counted, contributing to underestimation of the population [37, 38]. Underestimation of deaths among APIs may also occur if persons who spend most of their working life here in the US return to their home country for retirement and death.

## 5. Conclusions 

Asians are the fastest growing racial/ethnic group in the US, and they have a unique cancer burden that requires special attention. The leading cause of death in APIs nationally and in NYC is malignant neoplasms. APIs are disproportionately affected by some less common cancers, including nasopharyngeal, liver, and stomach cancers. Lung cancer rates among the Chinese, especially men, remain high.

Much of the API cancer burden in NYC is potentially preventable. Hepatitis B screening has been recommended by CDC for high-risk groups, including persons from China and Korea. Culturally tailored tobacco control policies and programs have been implemented over the last decade, and further evaluation is needed to determine their impact. In those populations that have reduced smoking rates, the impact may become more apparent in the next decade as fewer people develop smoking-related cancer, given that there is a long lag time between smoking exposure and cancer development. Potential population-based screening approaches to detect and treat early nasopharyngeal and stomach cancers in APIs require more evaluation before implementation but should be considered. The substantial variation in cancer-specific mortality rates across Asian racial subgroups indicates that API subgroup analyses such as these are necessary to plan public health interventions.

## Figures and Tables

**Table 1:** Decedent characteristics for selected cancer deaths^a^ by racial/ethnic groups in New York City, 2001–2010.

	Asian	White	Hispanic	Black	Total
Deaths	5,548	43,036	12,046	21,978	82,608
Males (%)	57.7	46.7	50.6	47.2	48.1
Average age, median	67.1 (68.0)	72.6 (74.0)	67.1 (68.0)	67.5 (68.0)	70.1 (71.0)
Education					
Less than high school	41.2	12.2	32.4	18.0	18.4
High school	28.3	48.8	52.0	52.8	48.9
Greater than high school	28.4	36.3	13.2	25.6	29.4
Education unknown	2.1	2.7	2.3	3.6	2.8
Married (%)	67.1	45.1	41.3	34.4	43.2

^a^Analysis limited to decedents who died from the cancers selected for study (i.e., lung, colon and rectum, esophagus, prostate, breast, ovary, cervix, nasopharynx, liver, and stomach).

**Table 2:** Age-adjusted cancer mortality rate (CMR) per 100,000 by racial/ethnic groups in New York City, 2001–2010.

	API		White		Hispanic		Black	
Cancer Type	CMR	(95%CI)	CMR	(95%CI)	CMR	(95%CI)	CMR	(95%CI)
Males								
All cancers								
Nasopharynx^a^	2.57	(2.06, 3.07)	0.29	(0.22, 0.39)	0.31	(0.20, 0.48)	0.48	(0.34, 0.66)
Esophagus	3.4	(2.7, 4.1)	5.9	(5.6, 6.3)	5.8	(5.1, 6.4)	7.6	(6.9, 8.3)
Liver	15.9	(14.6, 17.3)	7.9	(7.5, 8.3)	13.6	(12.7, 14.5)	11.6	(10.8, 12.4)
Stomach	11.8	(10.5, 13.0)	6.0	(5.6, 6.3)	8.6	(7.8, 9.4)	10.2	(9.4, 11.0)
Colon	14.8	(13.4, 16.3)	20.8	(20.1, 21.4)	18.4	(17.2, 19.6)	26.4	(25.1, 27.7)
Lung	38.2	(36.0, 40.5)	49.5	(48.4, 50.5)	33.5	(31.9, 35.1)	54.3	(52.5, 56.1)
Prostate	8.0	(6.9, 9.2)	18.7	(18.0, 19.3)	24.1	(22.6, 25.6)	49.2	(47.3, 51.1)

Females								
All cancers								
Nasopharynx^a^	0.67	(0.55, 0.94)	0.13	(0.08, 0.19)	0.13	(0.07, 0.23)	0.28	(0.19, 0.39)
Esophagus	0.8	(0.6, 1.2)	1.7	(1.5, 1.8)	1.2	(1.0, 1.4)	2.6	(2.3, 2.9)
Liver	5.4	(4.7, 6.2)	3.0	(2.8, 3.3)	4.9	(4.5, 5.4)	3.8	(3.4, 4.1)
Stomach	5.6	(4.9, 6.4)	3.5	(3.2, 3.7)	4.8	(4.3, 5.2)	5.5	(5.1, 6.0)
Colon	9.7	(8.7, 10.7)	14.4	(13.9, 14.9)	12.3	(11.5, 13.0)	18.0	(17.2, 18.8)
Lung	16.7	(15.4, 18.0)	32.5	(31.8, 33.2)	14.7	(13.9, 15.4)	29.0	(28.0, 30.0)
Breast	9.8	(8.9, 10.8)	25.3	(24.7, 26.0)	16.4	(15.6, 17.2)	29.7	(28.7, 30.6)
Ovary	4.6	(3.9, 5.2)	9.4	(9.0, 9.8)	5.1	(4.7, 5.6)	6.9	(6.4, 7.4)
Cervix	2.1	(1.7, 2.6)	1.9	(1.7, 2.1)	3.2	(2.9, 3.6)	5.1	(4.7, 5.5)

^a^All nasopharyngeal cancer mortality rates reported to hundredth decimal place for accuracy.

**Table 3:** Decedent characteristics for selected cancer deaths^a^ by racial/ethnic subgroups in New York City, 2001–2010.

	Chinese	Korean	Asian Indian	Filipino	Total
Deaths	3,703	596	499	333	5,131
Males (%)	61.2	55.4	51.3	41.7	58.3
Average age (median)	68.6 (71.0)	66.7 (67.0)	61.6 (62.0)	64.8 (64.0)	67.4 (69.0)
Education					
Less than high school	50.6	23.8	20.6	6.9	41.7
High school	26.9	33.1	43.5	19.5	28.7
Greater than high school	19.9	38.6	32.7	71.2	26.6
Education Unknown	2.7	4.5	3.2	2.4	2.9
Married (%)	61.2	63.9	68.1	60.7	67.6

^a^Analysis limited to decedents who died from the cancers selected for study (i.e., lung, colon and rectum, esophagus, prostate, breast, ovary, cervix, nasopharynx, liver, and stomach).

**Table 4:** Age-adjusted cancer mortality rates (CMR) per 100,000 by racial/ethnic subgroups in New York City, 2001–2010.

	Chinese		Korean		Asian Indian		Filipino	
Cancer Type	CMR	(95%CI)	CMR	(95%CI)	CMR	(95%CI)	CMR	(95%CI)
Males								
Nasopharynx^a^	4.52	(3.65, 5.40)	0.40	(0.01, 2.22)	b		0.50	(0.06, 1.89)
Liver	19.5	(17.7, 21.4)	25.3	(19.8, 31.9)	5.6	(3.5, 8.5)	6.5	(4.1, 9.8)
Stomach	12.9	(11.4, 14.5)	27.7	(21.9, 34.7)	5.0	(3.1, 7.8)	1.7	(0.5, 3.9)
Colon	16.1	(14.3, 18.0)	17.6	(12.9, 23.5)	9.0	(5.5, 13.8)	6.3	(3.9, 9.5)
Lung	47.1	(44.0, 50.2)	38.9	(31.4, 46.5)	11.3	(8.2, 15.2)	15.0	(11.2, 19.7)
Prostate	6.8	(5.6, 8.0)	5.6	(3.1, 9.4)	8.8	(5.1, 14.3)	9.5	(6.3, 13.8)
Esophagus	4.2	(3.3, 5.2)	3.1	(1.4, 5.8)	1.7	(0.4, 4.8)	0.7	(0.1, 2.6)

Females								
Nasopharynx^a^	1.22	(0.83, 1.73)	b		b		0.45	(0.05, 1.65)
Esophagus	0.7	(0.4, 1.1)	0.4	(0.1, 1.6)	1.1	(0.3, 2.8)	0.5	(0.1, 1.7)
Liver	6.0	(5.0, 7.0)	7.5	(5.3, 10.4)	2.9	(1.6, 5.0)	2.6	(1.3, 4.5)
Stomach	5.3	(4.4, 6.2)	11.0	(8.2, 14.4)	2.2	(1.1, 4.1)	2.6	(1.3, 4.5)
Colon	10.6	(9.3, 11.9)	9.0	(6.5, 12.1)	4.2	(2.5, 6.7)	5.2	(3.3, 7.8)
Lung	20.0	(18.2, 21.8)	12.9	(9.8, 16.6)	6.1	(4.0, 8.8)	8.4	(5.9, 11.6)
Breast	9.5	(8.3, 10.7)	7.2	(5.1, 10.0)	11.0	(8.5, 14.0)	13.4	(10.2, 17.2)
Ovary	4.2	(3.4, 5.0)	4.0	(2.4, 6.2)	4.8	(3.3, 6.7)	6.4	(4.3, 9.2)
Cervix	2.0	(1.5, 2.7)	2.0	(0.9, 3.6)	2.0	(1.1, 3.3)	2.0	(1.0, 3.8)

^a^All nasopharyngeal cancer mortality rates reported to hundredth decimal place for accuracy.

^b^Rate not displayed due to no deaths.

## References

[1] Hoeffel EM, Rastogi S, Kim MO, Shahid H (2012). *2010 Census Brief C2010BR-11: The Asian Population*.

[2] United States Census Bureau Newsroom: 2010 census shows America’s diversity. http://www.census.gov/newsroom/releases/archives/2010_census/cb11cn125.html.

[3] New York City Department of Health and Mental Hygiene Bureau of vital statistics. Summary of vital statistics 2010. http://www.nyc.gov/html/doh/downloads/pdf/vs/vs-population-and-mortality-report.pdf.

[4] Eheman C, Henley SJ, Ballard-Barbash R (2012). Annual report to the nation on the status of cancer, 1975–2008, featuring cancers associated with excess weight and lack of sufficient physical activity. *Cancer*.

[5] U.S. Cancer Statistics Working Group (2012). United States cancer statistics: 1999–2008 incidence and mortality web-based report. http://apps.nccd.cdc.gov/uscs/.

[6] Chen MS (2005). Cancer health disparities among Asian Americans. *Cancer*.

[7] McCracken M, Olsen M, Chen MS (2007). Cancer incidence, mortality, and associated risk factors among Asian Americans of Chinese, Filipino, Vietnamese, Korean, and Japanese ethnicities. *CA Cancer Journal for Clinicians*.

[8] Kwong SL, Chen MS, Snipes KP, Bal DG, Wright WE (2005). Asian subgroups and cancer incidence and mortality rates in California. *Cancer*.

[9] Freeman K, Zonszein J, Islam N, Blank AE, Strelnick AH (2011). Mortality trends and disparities among racial/ethnic and sex subgroups in New York City, 1990 to 2000. *Journal of Immigrant and Minority Health*.

[10] Fang J, Madhavan S, Alderman MH (1996). Cancer mortality of Chinese in New York City 1988–1992. *International Journal of Epidemiology*.

[11] Miller BA, Chu KC, Hankey BF, Ries LAG (2008). Cancer incidence and mortality patterns among specific Asian and Pacific Islander populations in the U.S. *Cancer Causes and Control*.

[12] World Health Organization (2004). *ICD-10: International Statistical Classification of Diseases and Related Health Problems*.

[13] Klein RJ, Schoenborn CA (2001). Age adjustment using the 2000 projected U.S. population. *Healthy People 2000 Statistical Notes*.

[14] Ma GX, Shive SE, Tan Y, Feeley RM (2004). The impact of acculturation on smoking in Asian American homes. *Journal of Health Care for the Poor and Underserved*.

[15] GLOBOCAN 2008 cancer fact sheet: lung cancer incidence and mortality in China in 2008. http://globocan.iarc.fr/.

[16] Li Q, Hsia J, Yang G (2011). Prevalence of smoking in China in 2010. *The New England Journal of Medicine*.

[17] Nir S (2012). For many Asian New Yorkers, smoking is still a way of life. *New York Times*.

[18] Cummings KM, Hyland A, Fix B (2006). Free nicotine patch giveaway program. 12-month follow-up of participants. *American Journal of Preventive Medicine*.

[19] Kim S (2012). Smoking prevalence and the association between smoking and sociodemographic factors using Korea national health and nutrition examination survey data, 2008 to 2010. *Tobacco Use Insights*.

[20] GLOBOCAN 2008 cancer fact sheet: nasopharyngeal cancer incidence and mortality in China in 2008. http://globocan.iarc.fr/.

[21] Chang ET, Adami H-O (2006). The enigmatic epidemiology of nasopharyngeal carcinoma. *Cancer Epidemiology Biomarkers and Prevention*.

[22] Jia W-H, Luo X-Y, Feng B-J (2010). Traditional Cantonese diet and nasopharyngeal carcinoma risk: a large-scale case-control study in Guangdong, China. *BMC Cancer*.

[23] Hildesheim A, West S, DeVeyra E (1992). Herbal medicine use, Epstein-Barr virus, and risk of nasopharyngeal carcinoma. *Cancer Research*.

[24] Cao S-M, Simons MJ, Qian C-N (2011). The prevalence and prevention of nasopharyngeal carcinoma in China. *Chinese Journal of Cancer*.

[25] American Cancer Society Learn about cancer: nasopharyngeal cancer. http://www.cancer.org/cancer/nasopharyngealcancer/detailedguide/nasopharyngeal-cancer-survival-rates.

[26] Merican I, Guan R, Amarapuka D (2000). Chronic hepatitis B virus infection in Asian countries. *Journal of Gastroenterology and Hepatology*.

[27] Altekruse SF, McGlynn KA, Reichman ME (2009). Hepatocellular carcinoma incidence, mortality, and survival trends in the United States from 1975 to 2005. *Journal of Clinical Oncology*.

[28] Centers for Disease Control and Prevention (2012). Surveillance for chronic hepatitis B virus infection—New York City, June 2008-November 2009. *MMWR Morbidity Mortality Weekly Report*.

[29] France AM, Bornschlegel K, Lazaroff J, Kennedy J, Balter S (2012). Estimating the prevalence of chronic hepatitis B virus infection—New York City, 2008. *Journal of Urban Health*.

[30] Centers for Disease Control and Prevention (2006). Screening for chronic hepatitis B among Asian/Pacific Islander population—New York City, 2005. *MMWR Morbidity Mortality Weekly Report*.

[31] Huang V, Johnson N, Rude E (2012). Preventing and managing hepatitis B. *City Health Information*.

[32] Kim KE (2003). Gastric cancer in Korean Americans: risks and reductions. *Korean and Korean-American Studies Bulletin*.

[33] Ward MH, Kilfoy B, Sinha R, Hollenbeck AR, Schatzkin A, Cross A (2011). Ingestion of nitrate and nitrite and risk of stomach cancer in the NIH-AARP diet and health study. *Epidemiology*.

[34] Yoshida M, Kondo K, Tada T (2010). The relation between the cancer screening rate and the cancer mortality rate in Japan. *Journal of Medical Investigation*.

[35] Breastcancer.org More Asian women being diagnosed with breast cancer. http://www.breastcancer.org/symptoms/new_research/20070928.jsp.

[36] Ho R, Muraoka M, Cuaresma C, Guerrero R, Agbayani A (2010). Addressing the excess breast cancer mortality in Filipino women in Hawaii through AANCART, an NCI community network program. *Hawaii Medical Journal*.

[37] Asian American Justice Center Advancing equality: the truth about Asian-Americans and the census: debunking the myths. http://www.advancingequality.org/attachments/wysiwyg/144/AAJCTruthAboutCensus.pdf.

[38] Pew Research Center The rise of Asian Americans. http://www.pewsocialtrends.org/files/2012/06/SDT-The-Rise-of-Asian-Americans-Full-Report.pdf.

